# The Chemists' Exhibition

**Published:** 1909-05-22

**Authors:** 


### THE CHEMISTS' EXHIBITION.

This Exhibition was held at the Horticultural Hall, estminster, S.W., on May 10 to 14 inclusive. Much edit is due to the organisers, "The British and Colonial uggist," for the arrangements and management, which lly provided for the convenience and comfort of visitors d exhibitors. Both in the interest of exhibits, and the [illegible]t and quality of attendance, the Exhibition was y successful. There was much to arrest attention a medical standpoint. In this respect the British Houses, Ltd., gave striking examples of what can be plished in inorganic chemicals; and Messrs. Arthur and Co., Ltd., the pill manufacturers, of Brighton, ted interest with their lactic tablets, Tholaform s, and hæmostatic Styptirenal. Messrs. Baiss, ers and Stevenson gave special prominence to their ver oil preparations; while Messrs. Parke, Davis and [illegible] Lucryl, Ltd., and Thomas Christy and Co. had extenve displays of toilet preparations.

Natural mineral waters were a feature of the Exhibition. Mention may be made of "Magi," a radio-active natural water, with distinctive therapeutic properties, shown by the Caledonia Springs Co., Ltd., and Friedrichshall water, often described in hepatic disorders. Messrs. Ingram and Royle, Ltd., invited attention to their Carlsbad water, Carlsbad Sprudel salts, and Cheltenham Spa water; while Messrs. Findlater and Co., who of late have devoted themselves to the importation of Continental mineral waters, had on view the Wildungen, Martigny, and Carabaña waters. The Sanitas Co., Ltd., gave an extensive display of their various disinfectants, soaps, etc.; their sanitary floor polish for hospital use; and Sanitas-Bactox for medical and obstetrical work. Messrs. Howison and Co., manufacturers of surgical rubber goods, made a prominent feature of gloves for operations and post-mortem examinations. This firm might with advantage direct their energies to the supply of india-rubber goods to hospitals.

